# Antibacterial and Barrier Properties of Gelatin Coated by Electrospun Polycaprolactone Ultrathin Fibers Containing Black Pepper Oleoresin of Interest in Active Food Biopackaging Applications

**DOI:** 10.3390/nano8040199

**Published:** 2018-03-28

**Authors:** Kelly Johana Figueroa-Lopez, Jinneth Lorena Castro-Mayorga, Margarita María Andrade-Mahecha, Luis Cabedo, Jose Maria Lagaron

**Affiliations:** 1 Optoelectronics Group, Interdisciplinary Science Institute, Faculty of Basic Science and Technologies, Universidad del Quindío, Carrera 15 Calle 12 Norte, 630004 Armenia, Colombia; kjfigueroal@iata.csic.es; 2 Novel Materials and Nanotechnology Group, Instituto de Agroquímica y Tecnología de Alimentos (IATA), Calle Catedrático Agustín Escardino Benllonch 7, 46980 Valencia, Spain; 3 Nanobiotechnology and Applied Microbiology (NANOBIOT), Universidad de los Andes, 11711 Bogotá, Colombia; jincasma@iata.csic.es; 4 Group of Research on Agroindustrial Processes (GIPA), Universidad Nacional de Colombia, 763533 Palmira, Colombia; mmandradem@unal.edu.co; 5 Polymers and Advanced Materials Group (PIMA), Universitat Jaume I (UJI), Avenida de Vicent Sos Baynat s/n, 12071 Castellón, Spain; lcabedo@uji.es

**Keywords:** nanofibers, electrospinning coating technique, gelatin, polycaprolactone, antimicrobials

## Abstract

The present study evaluated the effect of using electrospun polycaprolactone (PCL) as a barrier coating and black pepper oleoresin (OR) as a natural extract on the morphology, thermal, mechanical, antimicrobial, oxygen, and water vapor barrier properties of solvent cast gelatin (GEL). The antimicrobial activity of the developed multilayer system obtained by the so-called electrospinning coating technique was also evaluated against *Staphylococcus aureus* strains for 10 days. The results showed that the multilayer system containing PCL and OR increased the thermal resistance, elongated the GEL film, and significantly diminished its permeance to water vapor. Active multilayer systems stored in hermetically closed bottles increased their antimicrobial activity after 10 days by inhibiting the growth of *Staphylococcus aureus*. This study demonstrates that addition of electrospun PCL ultrathin fibers and OR improved the properties of GEL films, which promoted its potential use in active food packaging applications.

## 1. Introduction

The environmental issues generated by the slow degradation rate of the plastics once discarded after use have fostered the study and development of biodegradable polymers to obtain continuous matrices for the production of biodegradable packaging [1]. Among the bio-based biodegradable polymers, gelatin is one of the proteins with the greatest industrial applications due to its gelling properties, ability to form and stabilize emulsions, its adhesive properties, and dissolution behavior [2]. This biopolymer has a unique sequence of amino acids with a high content of proline, glycine, and hydroxyproline, which help in the formation of flexible films [3] and presents a good barrier against oxygen and carbon dioxide. For these reasons, gelatin is a very promising candidate for new bio-based and biodegradable packaging formulations. However, gelatin is very sensitive to moisture and, therefore, is not an effective barrier against water vapor. In this regard, researchers have worked on improving gelatin properties by blending it with other moisture resistant biodegradable polymers [4]. However, the multilayer structures are an alternative for improving the performance of biopolymers since it is the most efficient disposition to yield barrier properties [5,6]. In the present work, the use of a multilayer approach was developed to impart water resistance to GEL films. 

One of the biodegradable aliphatic polyesters with more applications and easier processing is polycaprolactone (PCL). It poses viscoelastic properties superior to other polyesters [7], which is compatible with polymers from renewable sources and it allows the formation of multilayer structures. PCL is hydrophobic and, therefore, is an alternative for reducing hydrophilicity of gelatin and improve the water vapor permeability [8]. Most studies of PCL with gelatin using the electrospinning technique have focused on tissue engineering applications and have never been related to multilayers [9,10,11,12,13]. 

In order to obtain the multilayer structure, the PCL layer will be deposited onto the gelatin films by electrospinning. Electrospinning is a technique that allows the design of materials and structures with improved properties due to their ability to create nano and micro-scale structures with variable fiber diameters and porosity [14]. This technique involves applying electrical forces that exceed the surface tension of viscoelastic polymer solutions. The morphology and diameter obtained in fibers or spheres are influenced by the type of polymer, viscosity, electrical conductivity, polarity, surface tension, and operational conditions of the equipment. The advantages of this technique is that it runs at room temperature, which avoids the loss of antimicrobial and antioxidant activity of encapsulated compounds [15,16]. The technique has more recently been used to coat materials such as paper and polymers [17,18,19]. 

Finally, the development of active packaging systems is the most promising strategy to extend shelf food life and safety. There are several compounds obtained from natural sources such as essential oils and oleoresins, which have active compounds. This makes them interesting for use as antimicrobials and antioxidants [20,21]. In the food industry, the use of these natural compounds has been intensified since most of the antimicrobial agents available are chemically synthesized. Moreover, in some cases, they have been banned in different countries because they are linked to several health problems [1,22]. The active compounds such as essential oils and oleoresins have been incorporated into the polymer matrices in order to provide the biodegradable material with active properties and, at the same time, improve the mechanical and barrier properties due to their hydrophobic character. Some authors have shown that the essential oils incorporated in gelatin films impact on the molecular structure affects the thermal properties of the material and causes a decrease in water vapor permeability and tensile strength, which increases elongation [5,6,22,23,24].

Water vapor permeability and antimicrobial performance are essential properties for controlling packaging materials because water molecules promote biochemical and microbiological reactions that can deteriorate food quality and even safety [23]. In this study, multilayer systems based on gelatin and PCL containing black pepper oleoresin were developed for the first time using the electrospinning coating technique in order to impart gelatin with antimicrobial properties and water vapor barrier performance. The morphology, mechanical, thermal, and barrier properties as well as antimicrobial capacity against strains of *S. aureus* of the materials were evaluated.

## 2. Materials and Methods 

### 2.1. Materials 

To elaborate the films, food-grade gelatin was used with a bloom of 220–240 g (Gelco S.A., Barranquilla, Colombia) along with pharmaceutical-grade microcrystalline cellulose, Avicel**^®^** PC 105 (FMC Biopolymer, Campinas, Brazil); 99% purity glycerol (Sigma Aldrich, St. Louis, MI, USA); Tween 80 for synthesis (Merck, Darmstadt, Germany); and black pepper oleoresin (TECNAS S.A., Antioquia, Colombia). For the coating systems, Poly (ε-caprolactone) (PCL) (Mn = 80,000) (Sigma Aldrich, Madrid, Spain), chloroform (Sigma Aldrich), and butanol (Sigma Aldrich) were used without further purifications steps. 

### 2.2. Elaboration of the Gelatin Films by Solution Casting 

The film-forming solution (FFS) was obtained from an aqueous suspension of microcrystalline cellulose (0.15 g/100 g of FFS) magnetically agitated for 30 min at 35 °C. Simultaneously, two aqueous suspensions were also prepared, which involves stirring gelatin (3 g/100 g of FFS) at 60 °C for 30 min and stirring another suspension containing glycerol (0.45 g/100 g of FFS) with agitation at 35 °C for 15 min. Afterward, all the components were mixed and kept at 60 °C for 15 min under constant agitation. The gelatin solution was cast onto plastic plates to obtain ~62 μm thick films after solvent evaporation in a laboratory fume hood at room temperature for 24 h. Finally, the gelatin films were removed from the plastic plates.

### 2.3. Preparation of the Water Barrier and Active Solution

The multilayer system was developed by depositing a PCL fibers layer onto both sides of the gelatin film. A PCL solution was prepared by dissolving 10% (wt/vol) of PCL in a chloroform/butanol 75:25 (vol/vol) mixture at room temperature. Black pepper oleoresin was incorporated into the solution at 7% in weight (wt %).

### 2.4. Preparation of the Electrospun Coatings

**Figure 1:**
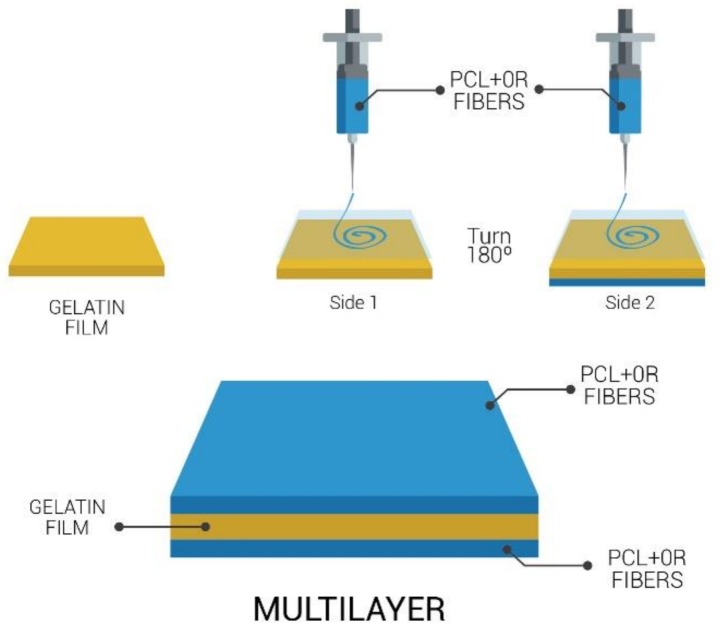
Scheme of the multilayer system (PCL+OR)-GEL-(PCL+OR).

The PCL fibers containing black pepper oleoresin were directly electrospun onto both sides of the gelatin films using a high throughput electrospinning/electrospraying pilot line Fluidnatek^®^ LE 500 manufactured and commercialized by Bioinicia S.L. (Valencia, Spain). The solutions were electrospun under a constant flow using a 24 emitter multi nozzle injector that scans vertically onto the gelatin film (see Figure 1). A voltage of 20 kV, a flow-rate of 1.5 mL/h per single emitter, and a tip-to-collector distance of 18 cm was used. A PCL coating without black pepper oleoresin was used as control. The electrospun conditions were different for the latter materials including an applied voltage of 30 kV, a flow-rate of 1 mL/h per single emitter, and a tip-to-collector distance of 18 cm were used. To obtain transparent, adhesive, and continuous coating layers based on PCL, a curing heating step was applied. To do so, the multilayers were placed between hot plates without applying pressure at 70 °C for several seconds in order to obtain self-adhering ultrathin fibers coalescence and a continuous PCL coating. The various monolayer and multilayer structures developed in the study with the corresponding coding used throughout the paper are gathered in Table 1. 

### 2.5. Characterization of the Materials 

#### 2.5.1. Film Thickness

Before testing, the thickness of all the structures was measured using a digital micrometer (S00014, Mitutoyo, Corp., Kawasaki, Japan) with ±0.001 mm accuracy. Measurements were performed and averaged in five different points with two in each end and one in the middle.

#### 2.5.2. Morphology 

The morphology of the PCL electrospun fibers and multilayer films was examined with scanning electron microscopy (SEM). The SEM micrographs were taken using a Hitachi S-4800 electron microscope (Tokyo, Japan) at an accelerating voltage of 10 kV and a working distance of 8–10 mm. The samples were previously sputtered with a gold-palladium mixture for three minutes under vacuum. The average fiber diameter was determined via ImageJ software (National Institutes of Health, Bethesda, MD, USA).

#### 2.5.3. Transparency

The light transmission of the samples was determined in specimens of 50 × 30 mm by quantifying the absorption of light at wavelengths between 200 nm and 700 nm and using an UV–Vis spectrophotometer (VIS3000, Dinko, Instruments, Barcelona, Spain). The transparency value (T) was calculated using the Equation (1) below.

(1) $$T=\frac{A_{600}}{L}$$

where $A_{600}$ is the absorbance at 600 nm and L is the film thickness (mm) [25].

#### 2.5.4. Thermogravimetric Analysis (TGA)

The thermogravimetric analysis of GEL films, PCL fibers, and multilayer structures was performed under nitrogen atmosphere in a Perkin Elmer Thermobalance TGA 7 (Perkin Elmer, Waltham, MA, USA). TGA curves were obtained after conditioning the sample in the sensor for 5 min at 30 °C. Then the samples were heated from 30 °C to 600 °C at a rate of 10 °C/minute. Derivative TGA curves (DTG) express the weight loss rate as a function of temperature and they were obtained using TA analysis software. All tests were carried out in triplicate. 

#### 2.5.5. Water Vapor Permeance (WVP)

The WVP of GEL films, PCL, and multilayer structures was determined according to the ASTM [26] gravimetric method using Payne permeability cups (Elcometer SPRL, Hermelle/s, Lieja, Belgium) of 3.5 cm diameter. One side of the films was exposed to 100% relative humidity (RH) by avoiding direct contact with liquid water. Then the cups containing the films were secured with silicon rings and stored in a desiccator at 25 °C and 0% RH. The cups were weighed periodically after the steady state was reached. Measurements were done in triplicate for each type of samples. WVP was calculated from the steady-state permeation slopes obtained from the regression analysis of weight loss data over time as previously reported [23]. The data was corrected for weight loss through the sealing of the OR. 

#### 2.5.6. Water Contact Angle

The films’ surface wettability was evaluated by using dynamic water contact angle (WCA) measurements in an Optical Tensiometer (Theta Lite, Staffordshire, UK). Five droplets (5 µL·s^−1^) were seeded on the surfaces of three samples of each studied material with the size of 2 × 5 cm^2^ and the resulting average contact angle was calculated.

#### 2.5.7. Oxygen Permeance 

The oxygen permeance (OP) measurements were recorded using an Oxygen Permeation Analyzer M8001 (Systech Illinois, Thame, UK) at 80% RH and 23 °C. A sample of each multilayer film (5 cm^2^) was placed in the test cell. The samples were previously purged with nitrogen in the humidity equilibrated samples before exposure to an oxygen flow of 10 mL·min^−1^. The measurements were duplicated.

#### 2.5.8. Mechanical Test

Tensile mechanical measurements were performed according to ASTM Standard D 638 [27] on an Instron Testing Machine (Model 4469; Instron Corp; Canton, MA, USA). The samples were dumbbell-shaped. The crosshead speed was fixed at 10 mm/minute. Four samples were tested for each material and the average values of the mechanical parameters and standard deviations were reported. Tensile Modulus (E), Tensile Strength at Yield ($\sigma_{y}$), and Elongation at Break ($\varepsilon_{b}$) were calculated from the stress–strain curves and estimated from force–distance data.

#### 2.5.9. Antimicrobial Activity

The antimicrobial performance of the multilayer films was evaluated by using a modification of the Japanese Industrial Standard JIS Z2801 (ISO 22196:2007). The *S. aureus* strain CECT240 (ATCC 6538p) was obtained from the Spanish Type Culture Collection (CECT: Valencia, Spain) and stored in phosphate buffered saline (PBS) with 10 wt % tryptic soy broth (TSB, Conda Laboratories, Madrid, Spain) and 10 wt % glycerol at −80 °C. Previous to each study, a loopful of bacteria was transferred to 10 mL of TSB and incubated at 37 °C for 24 h. A 100 µL aliquot from the culture was again transferred to TSB and grown at 37 °C to the mid-exponential phase of growth. The approximate count of 5 × 10^5^ CFU/mL of culture with an absorbance value of 0.20 as determined by optical density at 600 nm (Agilent 8453 UV–visible spectrum system, Deutschland, Germany). A microorganism suspension was applied on to the test (PCL+OR)-GEL-(PCL+OR) and PCL-GEL-PCL (negative control without OR) films of sizes 1.5 × 1.5 cm that were in hermetically closed bottles were applied. After incubation at 24 °C and at a relative humidity of at least 95% for 24 h, bacteria were recovered with PBS, 10-fold serially diluted, and incubated at 37 °C for 24 h in order to quantify the number of viable bacteria by conventional plate count. The antimicrobial activity was evaluated from one to 10 days. The value of the antimicrobial activity (R) was calculated by determining log10 ($N_{0}/N_{t}$) where $N_{0}$ is the average of the number of viable cells of bacteria on the untreated test piece after 24 h and $N_{t}$ is the average number of viable cells of bacteria on the antimicrobial test piece after 24 h.

#### 2.5.10. Statistical Analysis 

Each treatment was done in triplicate and the results of the properties were evaluated with a 95% significance level (*p* ≤ 0.05). Analysis of variance (ANOVA) and a multiple comparison test (Tukey) allowed to identify significant differences among the treatments. For this purpose, we used the software OriginPro8 (OriginLab Corporation, Northampton, MA, USA).

## 3. Results and Discussion

### 3.1. Morphology

**Figure 2:**
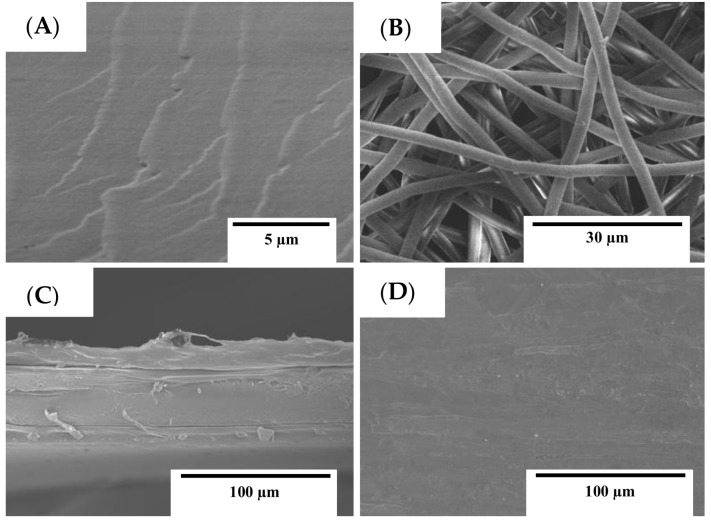
SEM pictures of (**A**) GEL film; (**B**) PCL fibers; (**C**) multilayer (PCL-GEL-PCL); and (**D**) multilayer surface.

The properties of the materials are significantly affected by their morphology. This means homogeneity, adhesion, and the structure of PCL, GEL, and active multilayers films were first assessed from SEM observations. Figure 2 shows the micrographs of the surface of the GEL film and PCL fibers as well as the surface and cross section of the multilayer film (PCL-GEL-PCL). The GEL film was seen to present homogeneous and smooth surfaces without visible pores or cracks (see Figure 2A). The PCL fibers presented uniform diameters with sizes of the order of ~2.5 µm (see Figure 2B). It was observed that the multilayers of PCL fibers on the GEL film produced a continuous surface that is expected to enhance the water barrier properties of the GEL film (see Figure 2C). The cross-sectional micrograph of the multilayer PCL-GEL-PCL presented a homogeneous and compact structure influenced by the annealing process (see Figure 2D). The multilayer system showed sufficient adhesion of the gelatin film to the PCL layers.

**Figure 3:**
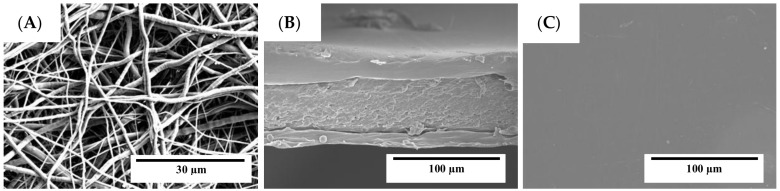
SEM pictures of (**A**) PCL fibers with OR; (**B**) active multilayer (PCL+OR)-GEL-(PCL+OR); and (**C**) active multilayer surface.

Figure 3A indicates that the PCL+OR fibers have smaller diameters (~0.7 µm) compared to the PCL fibers (see Figure 2B), which were also seen to present a larger fiber size distribution. This indicates that the presence of the oil molecules (OR) interferes in the properties of the mixture such as viscosity, which produces fibers with smaller diameters [28,29]. In Figure 3B,C, images of the active multilayer (PCL+OR)-GEL-(PCL+OR) in its cross-section and surface after the curing process are presented [30]. The cross-sections shown in Figure 2C and Figure 3B were seen to exhibit a laminate-like structure in which homogeneous PCL based coatings are located on both sides of the GEL films. The adhesion between the outer layers of PCL+OR and the GEL film was superior and only a weak delamination occurred after criofracturing the material. The thickness of the multilayer films was the same (~80 µm). The active multilayer (see Figure 3C) presented a more homogeneous and continuous surface after annealing than the multilayer seen in Figure 2D. The OR seemed to promote good phase interaction with the PCL as expected.

**Figure 4:**
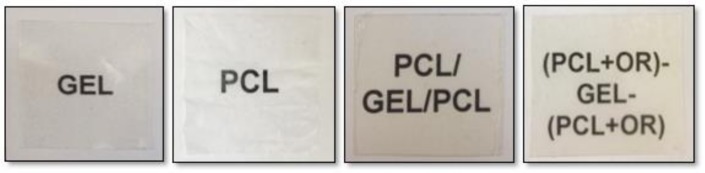
Contact transparency pictures, from left to right, GEL, PCL, PCL-GEL-PCL, and (PCL+OR)-GEL-(PCL+OR) films.

Figure 4 gathers contact transparency pictures of the GEL film and of the multilayer systems. The GEL film was seen to be the most transparent sample and presented a transparency (T) value as determined by Equation (1) while the PCL was the material with the lowest transparency, which presented a T value of 13.1. The multilayer systems presented intermediate transparency values. For example, PCL-GEL-PCL presented a T value of 5.2 whereas (PCL+OR)-GEL-(PCL+OR) presented a value of 5.6. Even though the multilayer systems presented lower transparency than GEL as expected, the contact transparency pictures in Figure 4 indicate that they are not opaque so the content of the packaging can still be assessed. From a positive viewpoint, light penetration prevention especially in the UV region can also help reduce photoxidation of organic compounds present in foods [31].

### 3.2. Thermogravimetric Analysis

**Figure 5:**
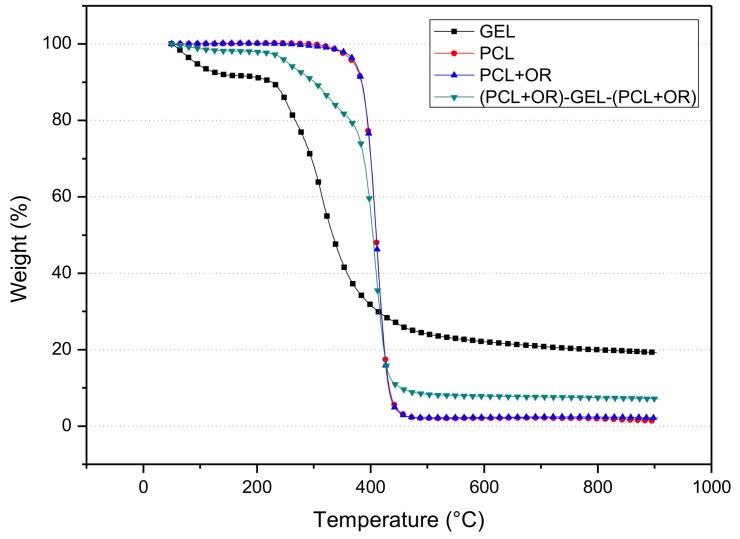
TGA curves of GEL, PCL, PCL+OR, and (PCL+OR)-GEL-(PCL+OR) films.

Understanding how the thermal properties of materials are affected by additives is paramount to potentially screen packaging failure during processes such as hot filling, humid heat sterilization, and melt compounding. From a fundamental viewpoint, they also tell something about intercomponent interactions and thermal reactivity [32,33]. It is clear that our coating technology avoids the use of temperature to preserve the antimicrobial additive stability and function but it is still of interest to see how PCL and OR affect the overall stability of one another and of GEL. Therefore, TGA experiments were carried in the various samples and the thermograms are gathered in Figure 5. From the figure, the films were seen to exhibit a thermal decomposition process with onset at ca. 240 °C for the GEL film, 370 °C for the (PCL+OR)-GEL-(PCL+OR) film, and 350 °C for the PCL and PCL+OR films. 

The mass loss of the GEL film occurred in three stages. The first stage is attributed to the loss of moisture, and it occurs from 50 °C to about 200 °C. The second stage of the mass loss corresponds to the main thermal degradation zone that takes place between 200 °C and 415 °C where the peptide bonds break and the third stage was around 450 °C, which corresponds to the thermal decomposition of the GEL. Interestingly, the addition of OR to the PCL was not seen to alter the thermal stability of the PCL. 

In the thermograms of the PCL-GEL-PCL and (PCL+OR)-GEL-(PCL+OR) films, the two polymers were seen to contribute their characteristic thermal behavior. The first mass loss stage is then associated with the loss of water from GEL. The second stage indicates the decomposition of GEL and the third stage corresponds to the decomposition of PCL. The GEL film is then seen to increase its thermal stability by incorporating the active coating.

### 3.3. Water Vapor Permeance and Oxygen Permeance

Table 2 gathers the results of the water vapor permeance and oxygen permeance of the GEL, PCL, PCL+OR, and (PCL+OR)-GEL-(PCL+OR) films. Since the PCL is an aliphatic polyester, it is a hydrophobic material that provides water barrier performance to hydrophilic materials. Therefore, the addition of PCL was aimed at decreasing the water vapor transmission rate of the GEL film as expected. The water permeance of the gelatin films decreased significantly from 2290 × 10^−10^ Kg·m^−2^·s^−1^·Pa^−1^ to 3.34 × 10^−10^ Kg·m^−2^·s^−1^·Pa^−1^ for PCL-GEL-PCL films and 9.43 × 10^−10^ Kg·m^−2^·s^−1^·Pa^−1^ for (PCL+OR)-GEL-(PCL+OR) films. This reduction in the water permeance is expected due to the hydrophobic character of the PCL, which prevents the sorption and diffusion of water molecules [13] and, therefore, increases the hydrophobicity of the multilayer. A small permeability increase was observed after adding OR due to the oily additive imposing a less continuous morphology, which may lead to preferential paths at the polymer-additive interphase for the transport of water molecules [15]. The results are in agreement with previous studies that demonstrated the multilayer strategy can help significantly reduce the water vapor permeance of films made of hydrophilic polymers [29,34]. 

The oxygen permeance values of the multilayer with PCL and of the neat GEL did not present significant differences (see Table 2). The GEL film has a very high barrier to oxygen (13.8 ± 1.7 × 10^−15^) when compared to that of bio polyesters [4]. The OP values for such thin electrospun PCL films were over-ranged because permeation for this bio polyester is very high, which was reported earlier [35,36]. Due to this fact, the oxygen permeance of the multilayer (8.2 ± 1.1 × 10^−15^) did not show significant difference when compared with the neat GEL film as would be expected. The multilayer with PCL+OR was not tested because the potential release of organic vapors from the OR additive could interact and/or damage the redox detector of the permeation equipment. So the overall results indicate that the multilayer technology developed here did not alter the very high barrier of neat GEL to oxygen but it reduced to a significant extent the water barrier of the neat GEL. 

### 3.4. Water Contact Angle 

**Figure 6:**
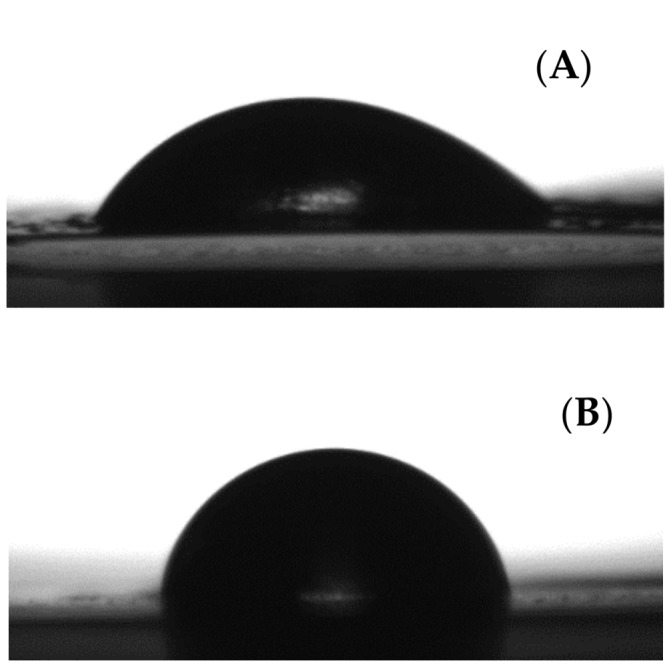

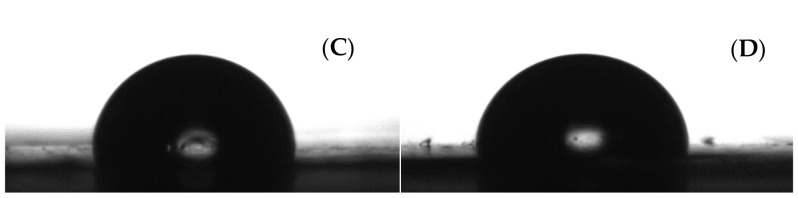
Images of water droplets in contact angle measurements of the GEL film (**A**); PCL film (**B**); multilayer systems PCL-GEL-PCL (**C**); and (PCL+OR)-GEL-(PCL+OR) (**D**).

Table 3 and Figure 6 gather the contact angle results carried out on the films. As expected, the contact angle of the multilayers was significantly higher than that of GEL, which suggests the efficient coating of this with PCL. Therefore, the GEL film presented a contact angle of ca. 50°, which is characteristic of hydrophilic materials and is in agreement with previous works [37,38]. The PCL film was seen to present a contact angle of ca 74°. In the case of the multilayers PCL-GEL-PCL (83.9°) and (PCL+OR)-GEL-(PCL+OR) (85.6°), the values were seen even higher compared to the values of the two neat materials. Therefore, these results clearly indicate that the wettability of the multilayers is reduced compared to the neat GEL as expected. The reason why the wettability is higher than that of electrospun fibers may be related to the different surface topology that could result from the multilayer formation. The observed enhanced water resistance is a positive result for the potential application of these multilayers in food packaging applications of moisture containing products such as sliced bread [39]. 

### 3.5. Mechanical Test 

The mechanical properties of the films are gathered in Table 4. From this table, the GEL film is seen to be clearly influenced in its mechanical performance by coating with PCL and PCL+OR. Therefore, the neat GEL film was seen to have higher values of tensile modulus and tensile strength as expected. For the elongation at break, the results were different. After coating the GEL-based multilayer, it reached higher elongated rates due to both the inherently higher plasticity of the PCL polymer and the high interfacial adhesion between layers. As a result, the mechanical properties of the films were strongly influenced by the interlayer interaction and design as previously reported [40,41].

### 3.6. Antimicrobial Activity 

**Figure 7:**
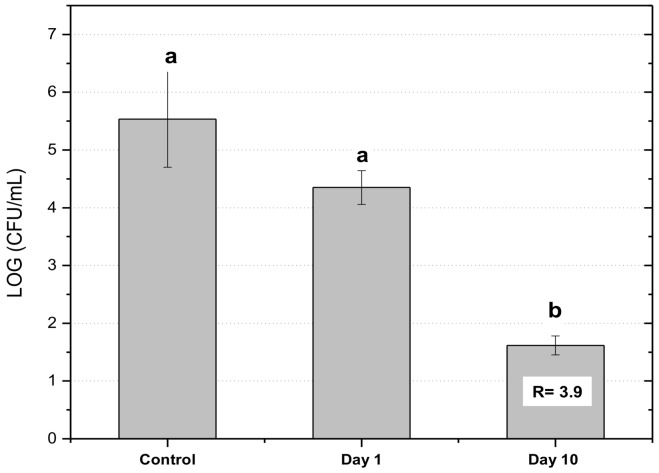
Antimicrobial activity of (PCL+OR)-GEL-(PCL+OR) film on 1 and 10 days’ storage against *S. aureus* CECT 240 after 24 h exposure. Different letters (a-b) indicate significant differences (*p* < 0.05) among samples. R correspond to the value of the antimicrobial activity.

*S. aureus* is one of the most common microorganisms associated with food intoxication. Therefore, the design of packaging materials with active properties is important from a food safety viewpoint [42]. The antimicrobial activity of multilayer films (PCL+OR)-GEL-(PCL+OR) against strains of *S. aureus* is presented in Figure 7. The control used in the analysis (PCL-GEL-PCL) presented a growth of 5.58 CFU/mL. In the first day of storage, the multilayer films containing OR showed a growth of 4.37 CFU/mL, which indicates 1 Log units of reduction with respect to the control. On day 10 of storage, the growth of *S. aureus* was 1.58 CFU/mL. This showed a reduction of 4 Log units (3 Log CFU/mL is the minimal value that a material can present to be considered as an antimicrobial [43,44]), with significant differences compared to the control and the first day sample (*p* ˂ 0.05). Over time there is an increase in the inhibition of the strain due to the greater release of active compounds present in the OR such as piperine, trans-β-caryophyllene, and limonene [45,46]. According to the existing literature, the mechanism of action consists of the antimicrobial species attack to the bilayer of phospholipids of the cellular membrane, which exposes the genetic material of the bacteria and generates the oxygenation of unsaturated fatty acids that form hydroperoxidase [1]. 

## 4. Conclusions

Multilayer systems made of ultrathin electrospun fibers were developed with the aim of enhancing the water resistance and mechanical performance of high oxygen barrier GEL films of interest in active food packaging applications. More specifically, GEL cast films were coated by the so-called electrospinning coating technique with PCL and PCL+OR. The multilayers reduced wettability and enhanced water barrier and elongation at break. Lastly, the addition of the OR bioactive component to the coating was seen to provide a strong antimicrobial behavior against *S. aureus*. The multilayer was also seen to improve the antimicrobial activity over time, which resulted in a controlled release of the antimicrobial active components for at least the first 10 days after processing.

## Figures and Tables

**Table 1:** Sample coding.

Sample Code	Composition
GEL	Gelatin film
PCL	Polycaprolactone fibers mats after curing
PCL-GEL-PCL (PCL+OR)-GEL-(PCL+OR)	Multilayer system Active multilayer system containing 7 wt % of black pepper oleoresin (OR)

**Table 2:** Sample thickness, water vapor, and oxygen permeance of GEL, PCL, PCL-GEL-PCL, and (PCL+OR)-GEL-(PCL+OR) films.

Sample Code	Thickness (µm)	WVP × 10^−10^ (Kg·m^−2^·s^−1^·Pa^−1^)	OP × 10^−15^ (m^3^·m^−2^·s^−1^·Pa^−1^)
GEL	60 ± 0.05	2290 ± 0.2 ^a^	13.8 ± 1.7 ^a^
PCL	17 ± 0.09	2.3 ± 0.5 ^b^	-
PCL-GEL-PCL	76 ± 0.3	3.3 ± 0.3 ^b^	8.2 ± 1.1 ^a^
(PCL+OR)-GEL-(PCL+OR)	79 ± 0.2	9.4 ± 0.7 ^b^	-

^a−b^ Different superscripts within the same column indicate significant differences among samples (*p* < 0.05).

**Table 3:** Contact angle values of the GEL, PCL, PCL-GEL-PCL, and (PCL+OR)-GEL-(PCL+OR) films.

Sample Code	θ (°)
GEL	50.3 ± 6.4 ^c^
PCL	74.3 ± 3.2 ^b^
PCL-GEL-PCL	83.9 ± 1.8 ^a^
(PCL+OR)-GEL-(PCL+OR)	85.6 ± 2.4 ^a^

^a−c^ Different superscripts within the same column indicate significant differences among samples (*p* < 0.05).

**Table 4:** Tensile parameters (E : Tensile Modulus, $\sigma_{y}$: Tensile Strength at yield and $\varepsilon_{b}$: Elongation at Break) of Gelatin, PCL-Gel-PCL, and (PCL+OR)-GEL-(PCL+OR) films.

Sample Code	E (MPa)	$\sigma_{y}$ (MPa)	$\varepsilon_{b}$ (%)
GEL	1392 ± 220 ^a^	42 ± 4.3 ^a^	5.83 ± 1.69 ^b^
PCL-GEL-PCL	883 ± 131 ^b^	31.2 ± 2.2 ^b^	15.5 ± 0.2 ^a^
(PCL+OR)-GEL-(PCL+OR)	745 ± 197 ^b^	29.7 ± 8.4 ^b^	17.4 ± 3.95 ^a^

^a,b^ Different superscripts within the same column indicate significant differences among samples (*p* < 0.05).
